# The State of the Heart in Fever

**Published:** 1855-07

**Authors:** 


*The State of the Heart in Fever.* By Dr. Stokes.

I have sometimes observed that students were under a misapprehension about the doctrines which we have long held in the hospital, with respect to the condition of the heart as a guide for the use of wine. They have come to the erroneous opinion that we are only to give wine where we find the want of the first sound of the heart, and that we are not to give wine where the heart is acting well. This is a mistaken view of the matter. What we have established as to the state of the heart in connection with the effect of stimulants, is simply this: we have ascertained that the efficacy of stimulants is often directly as the debility of the heart. It has been also ascertained, that the power of bearing stimulants, their effect upon the nervous system, their good effects on the general condition, are directly as the weakness of the heart. We may lay down as a rule, that there are three conditions of the heart to be looked at by the practical man in the treatment of fever. In one, we have an excited heart—a violently excited heart, all through the case; and this heart may be excited and violent, although the symptoms be those of extreme adynamia, although the surface be cold, the breath cold, and the pulse so feeble that it cannot be discovered. Nay, the heart may act with great force for several days, and yet there be no pulse at the wrist. This is one case. In the next case, we find exactly an opposite condition, in which the systolic force of the heart is diminished. This is shown by loss of impulse of the heart, by diminution of the first sound, and, in certain cases, by extinction of the first sound of the heart while the second remains. This is a case which calls for wine, and in which you should give it: it is a case in which, in the vast majority of instances, wine will agree with the patient. There is a third set of cases, in which the heart does not seem to be implicated at all in the course of the disease, in which, notwithstanding the existence of the most extraordinary group of symptoms affecting various organs, the heart, in the middle of the storm, seems to be in a state of calm and quiet. If we compare these three sets of cases, with a view to prognosis, we may arrange them in this way. The case of excited heart all through, with feeble pulse and with adynamia, is unquestionably the worst case. There is no worse symptom in fever than an excited heart. It is especially a bad symptom, when, with that of excitement, we find a feeble pulse. The next will be the case of sinking of the heart: and the most favorable case is that in which, as I said before, the heart seems to escape disease. But you are not to suppose, that because you have an excited heart you are not to give wine if the symptoms of the patient require it: and you are not to suppose that, because the heart is not affected at all, you are to withhold wine, if the general symptoms of the patient require it. You are not to found your exhibition of wine or stimulants upon any one thing; you are to take the general state of the patient into consideration. What we have done is to discover an intelligible, practical rule, which will guide you in the use of wine in certain, I think in many cases; but you are not to suppose that because this man has a clear first sound at his heart, therefore you are not to give wine. You are not to suppose that because the heart is safe, you can do without wine.—*Ranking's Abstract.*

